# A human-relevant alternative infection model for mucormycosis using the silkworm *Bombyx mori*

**DOI:** 10.1371/journal.pone.0333476

**Published:** 2025-09-25

**Authors:** Masaki Ishii, Kazuhiro Mikami, Fumiaki Tabuchi, Naho Maruyama, Atsushi Miyashita

**Affiliations:** 1 Research Institute of Pharmaceutical Sciences, Faculty of Pharmacy, Musashino University, Tokyo, Japan; 2 Institute of Medical Mycology, Teikyo University, Tokyo, Japan; 3 Graduate School of Medical Care and Technology, Teikyo University, Tokyo, Japan; 4 Department of Health and Dietetics, Faculty of Health and Medical Science, Teikyo Heisei University, Tokyo, Japan; East China Normal University School of Life Sciences, CHINA

## Abstract

Mucorales fungi cause life-threatening mucormycosis in patients with clinical risk factors, such as immunodeficiency. Moreover, they are resistant to several antifungal drugs, highlighting the urgent need for novel therapies. Traditional mammalian models are expensive and raise ethical concerns, thereby limiting their suitability for large-scale studies. We established a silkworm (*Bombyx mori*) infection model to investigate the pathogenicity of Mucorales and evaluated its relevance to human infection. Strains of *Rhizopus arrhizus*, *Mucor circinelloides*, and *Cunninghamella bertholletiae* induced fatal infections in the silkworm. Grocott-stained silkworm tissue sections revealed hyphal invasion patterns closely resembling those observed in human mucormycosis. In addition, experimental simulation of clinical risk factors (steroid use and iron overload) significantly reduced the median lethal dose (LD_50_). Treatment with the antifungal drug isavuconazonium prolonged the survival of *R. arrhizus*-infected silkworms, suggesting the potential use of this model for novel antifungal screening. LD_50_ estimation for four *R. arrhizus* strains revealed up to a 100-fold difference in pathogenicity to the silkworm among the tested strains, corresponding to variations in cell surface characteristics. Interestingly, the presence of high-molecular weight (50–100 kDa) cell surface proteins was associated with high pathogenicity among *R. arrhizus* strains. In conclusion, the silkworm is a viable, human-relevant alternative model to investigate Mucorales infections, with potential for large-scale antimucormycosis drug screening.

## Introduction

Mucormycosis is a life-threatening opportunistic infection caused by various fungal species within the order Mucorales, such as *Rhizopus*, *Rhizomucor*, and *Mucor* species. This disease is characterized by rapid and aggressive tissue invasion, which leads to necrotizing infections that can affect various anatomical sites, including the nasal sinuses and palate (rhinocerebral form), lungs (pulmonary form), skin and soft tissues (cutaneous form), gastrointestinal tract, and other organs. Mucormycosis is associated with high morbidity and mortality, with reported overall mortality rates of approximately 44%–54% following disease onset [1,2]. The major risk factors for mucormycosis include poorly controlled diabetes mellitus (particularly in the presence of ketoacidosis), hematological malignancies, prolonged corticosteroid use or other forms of immunosuppression, iron overload, deferoxamine therapy, and trauma or burns [3–6]. Although some mechanisms underlying the host environment–dependent increase in the pathogenicity of Mucorales have been proposed [7,8], several aspects remain unclear. In addition, unlike most clinically relevant fungal infections caused by Ascomycota (e.g., *Candida* and *Aspergillus*) and Basidiomycota (e.g., *Cryptococcus* and *Trichosporon*), mucormycosis is caused by Mucorales fungi belonging to the phylum Zygomycota. These fungi differ from other pathogenic fungi in terms of cellular characteristics and antifungal target molecule structures; moreover, they exhibit natural resistance to existing antifungal agents effective against non-Mucorales species [9,10]. Consequently, current antifungal therapies are limited to a few azole antifungals, such as isavuconazole and its prodrug isavuconazonium, and polyene macrolides with critical adverse effects, underscoring the need for new antifungal drugs.

In antifungal development, mammalian infection models have been instrumental for elucidating infection mechanisms and identifying antifungal candidates [9–12]. However, ethical concerns and high costs make large-scale animal experiments, such as genome-wide mutant screenings for pathogenic factors of fungal pathogens and high-throughput drug screening, impractical, necessitating alternative infection models. The silkworm *Bombyx mori*, a domesticated insect species historically used in sericulture [11], has emerged as a practical alternative infection model for studying not only fungi but also bacterial and viral pathogens [12–16]. In silkworm-based infection models, clinically relevant opportunistic pathogens can induce fatal infections [17–19]. Utilization of such models has facilitated the development of novel antimicrobials with unique mechanism of action, including lysocin E (effective against methicillin-resistant *Staphylococcus aureus* and drug-resistant *Mycobacterium tuberculosis*) and ASP2397 (effective against the lethal pathogenic fungus *Aspergillus fumigatus*) [20–22].

In this study, we sought to extend the application of silkworm-based alternative infection models to mucormycosis. In particular, we established a Mucorales infection model using *B. mori* and evaluated its relevance to human infections to assess its potential for antimucormycosis drug screening. This study challenges the prevailing paradigm that invertebrates are too distantly related to humans to serve as an effective human-relevant mucormycosis model. In addition to stimulating infection through inoculation with Mucorales spores in the silkworm, we investigated whether human clinical risk factors (i.e., steroid use, elevated iron levels, and iron chelation therapy) increase silkworm susceptibility. Furthermore, we examined the therapeutic efficacy of the clinically used antifungal agent isavuconazonium in this model. Finally, to further explore the utility of this silkworm-based model for studying pathogenic molecular mechanisms, we analyzed the relationship between cell surface molecular components and virulence using four *R. arrhizus* strains. Our findings establish silkworm as a human-relevant mucormycosis model, providing a valuable tool for elucidating host–pathogen interactions and facilitating the development of novel antifungal strategies.

## Materials and methods

### Fungal strain and culture conditions

Isolates of Mucorales, including *Rhizopus arrhizus* IFM46105 (from soil), IFM64256 (from sputum), IFM61553 (from fungus ball of paranasal sinuses), and IFM63826 (from paranasal sinus); *Mucor circinelloides* IFM67541 (from sputum); *Rhizomucor pusillus* IFM63523 (from lung); and *Cunninghamella bertholletiae* IFM60601 (from lung BALF), were purchased from Chiba university Medical Mycology Research Center, Chiba University, Chiba, Japan. Spores were prepared by culturing Mucorales on modified 1/10 Sabouraud dextrose agar (0.2% Bacto peptone, 0.1% glucose, 0.1% KH_2_PO_4_, 0.1% MgSO_4_ 7H_2_O, 1.5% agar) at 28°C [23]. Suspensions of sporangiospores were prepared in saline containing 0.05% (w/v) Tween 80, passed through a 40-μm cell strainer, and then resuspended in saline.

### Silkworm rearing conditions

Silkworms (*Bombyx mori*, strain KINSYU × SHOWA) were reared as previously reported [24,25]. In brief, silkworm eggs were purchased from a local supplier (Ehime Sanshu, Ehime Prefecture, Japan), surface-sterilized using 4% formaldehyde and ethanol, and reared on an artificial diet (Silkmake 2S, purchased from Nihon Nosan [Kanagawa Prefecture, Japan]) at 27°C. Individual larvae were isolated at the onset of the fourth molt (end of fourth instar larval stage), fed 1.1 g/larva of artificial diet per larva on the next day (i.e., day 1 of the fifth instar), and used for experiments on the subsequent day (i.e., day 2 of the fifth instar), unless otherwise stated.

### Infection experiment

A dilution series of spore suspensions was prepared from stock suspensions (2 × 10^8^ spores/mL) for *M. circinelloides* (IFM67541), *C. bertholletiae* (IFM60601), and *R. arrhizus* (IFM46105, IFM64256, IFM61553, and IFM63826) using physiological saline (Otsuka, product #14900AMZ00188). For each fungal strain, five to six serial dilutions were prepared, typically in the range of 10–100,000 spores/mL, corresponding to 0.5–5000 spores per larva when 50 µL was injected into larva. Each dilution was prepared in a 50-mL plastic centrifuge tube (Corning, product #352070) to a total volume of 10 mL. Fifth instar silkworm larvae (day 2) were injected with 50 µL of spore suspension into the hemolymph using a 1-mL disposable plastic syringe fitted with a 27-gauge needle (Terumo). For each strain, 10–20 larvae per concentration group were used to generate dose–response curves, and the median lethal dose (LD₅₀) was determined. Since microorganisms can adhere to the surfaces of plastic or glass containers [26,27], we confirmed that the materials of the containers used for preparing the dilution series and the syringe used for injection (i.e., whether plastic or glass) did not affect the outcomes of the infection experiments (Table S1). After inoculation, larvae were incubated at 27°C and monitored for mortality every 24 h over 72 h. The 72-h endpoint was selected as the cutoff for LD₅₀ determination based on preliminary experiments, as the majority of deaths occurred within this timeframe.

### Preparation and observation of silkworm tissue sections

Approximately 5,000 spores of *R. arrhizus* IFM46105 (in 50 µL of saline) were injected into the hemolymph of silkworm larvae. Following inoculation, the larvae were incubated at 27°C. The infected tissues were fixed by injecting 200 µL of 4% paraformaldehyde (Fujifilm Wako Pure Chemical Corporation, Tokyo, Japan, product #163–20145) into the hemolymph, followed by immersion in 25 mL of 4% paraformaldehyde for 2 weeks [28]. After fixation, they were outsourced to Sapporo General Pathology Laboratory Co., Ltd. (Hokkaido, Japan) for tissue sectioning. Paraffin-embedded sections of formalin-fixed silkworms were prepared and mounted on glass slides. The sections were stained using the Grocott’s methenamine silver staining method [29], which highlighted fungal elements in tissues.

### Effect of clinical risk factors on the silkworm infection model

To evaluate the influence of clinical risk factors on infection outcomes, we pharmacologically manipulated host immunity and iron availability prior to fungal challenge. For immunosuppression, 50 µL of a 10-mg/mL betamethasone solution (Tokyo Chemical Industry Co., Ltd., Tokyo, Japan, product #378-44-9) was injected into the hemolymph according to a previously described protocol [30]. To modulate iron levels, 50 µL of either a 1-mM FeCl₂ solution (Fujifilm Wako Pure Chemical Corporation, product #095−00912) or a 1-mg/mL solution of an iron chelator—deferoxamine mesylate, deferasirox, or deferiprone (Tokyo Chemical Industry Co., Ltd., product #138-14-7, #201530-41-8, #30652-11-0, respectively)—was administered. Dosages were selected based on previously reported studies using *Galleria mellonella* and murine mucormycosis models [31,32]. Subsequently, silkworm larvae were challenged with spores of *R. arrhizus* (strains IFM46105, IFM64256, IFM61553, and IFM63826). A 50-µL spore suspension was injected into the hemolymph using a 1-mL plastic syringe fitted with a 27-gauge needle. Depending on the strain and treatment condition, the inoculum was in the range of 5–50,000 spores per larva, as quantified using a hemocytometer. Following inoculation, larvae were incubated at 27°C and monitored for survival over 72 h. For each treatment condition, 10 larvae were used per dose group. LD₅₀ values were calculated from the dose–response curves derived from survival data.

### Scanning electron microscopy (SEM) of spores

Spores (2 × 10⁶ per sample) of *R. arrhizus* strains IFM46105, IFM64256, IFM61553, and IFM63826 were collected as described above and fixed in phosphate-buffered 2% glutaraldehyde (Fujifilm Wako Pure Chemical Corporation, Tokyo, Japan) at 4°C after centrifugation at 15,000 *× g* for 5 min. The fixed spores were then fixed in 2% osmium tetroxide (Nisshin EM Co., Ltd., Tokyo, Japan) for 2 h in an ice bath. Then, the specimens were dehydrated in a graded ethanol series (graded EtOH [30, 50, 70, 80, 90, 95, 100, 100, and 100%] each 15 min) and dried via CO_2_ critical point drying. The dried specimens were coated with an osmium plasma ion coater (OPC-80; Nippon Laser & Electronics Lab. Aichi, JAPAN) and examined using SEM (JSM-7500F at 5 kV).

### Minimum inhibitory concentration (MIC) determination

The MIC of isavuconazole against *R. arrhizus* IFM46105 was determined according to the CLSI M38-A2 guideline for filamentous fungi with the modification of the concentration range to 1.4-22 μg/ml. Spores were suspended in MOPS-buffered RPMI 1640 medium (pH 7.0) and adjusted to 2.5 × 10³ spores per 200 µL. Two-fold serial dilutions of isavuconazole (Tokyo Chemical Industry Co., Ltd., product #I1188) were prepared in 96-well microtiter plates to yield a final concentration range of 1.4–22 μg/ml. The plates were incubated at 35°C for 24 h. MIC was defined as the lowest isavuconazole concentration that resulted in complete inhibition of visible fungal growth (100% inhibition) in accordance with the CLSI guideline [33].

### Therapeutic effect of antifungal agents

A 50-µL spore suspension of each *R. arrhizus* strain (IFM46105, IFM64256, IFM61553, and IFM63826) was injected into the hemolymph of silkworm larvae. The inoculum was adjusted to 5,000 spores per larva for IFM46105 and IFM63826 and to 50,000 spores per larva for IFM64256 and IFM61553. Immediately after infection, 50 µL of 5 mM isavuconazonium (Sigma-Aldrich, USA) in saline was administered into the hemolymph. A control group received isavuconazonium alone without any fungal spore suspension to assess drug toxicity; no mortality was observed in this group. The number of larvae used per group was as follows: IFM46105 (control: n = 21, treated: n = 18), IFM64256 (control: n = 17, treated: n = 18), IFM61553 (control: n = 21, treated: n = 18), IFM63826 (control: n = 31, treated: n = 28), and isavuconazonium-only control (n = 13). After inoculation, larvae were incubated at 27°C and monitored for survival for up to 4 days. Survival curves were generated as described in the Statistical analysis section.

### Electrophoresis of fungal surface proteins

Surface proteins were extracted using a previously reported method [34]. In total, 3.5–5.6 × 10^8^ spores were treated with 1 mL of extraction buffer (1% β-mercaptoethanol, 1 mM ethylenediaminetetraacetic acid, protease inhibitor cocktail [Nacalai Tesque, product #03969−21], 50 mM sodium phosphate [pH 7.4]) and shaken at 37°C for 30 min. The supernatant was obtained via centrifugation at 13,000 rpm (19,840 × *g*) for 3 min, and 1 mL of 20% trichloroacetic acid was added with thorough mixing, followed by incubation at room temperature for 30 min. The resulting precipitate was centrifuged at 20,000 × *g* for 5 min at 4°C, resuspended in 1 mL acetone, mixed, and centrifuged again at 20,000 × *g* for 5 min at 4°C. The final pellet was designated as the surface protein fraction. Protein concentration was determined using the Protein Assay Bicinchoninate Kit (Nacalai, product #06385−00). A 1.25-μg aliquot of protein was separated via SDS-polyacrylamide gel electrophoresis. The gel was stained using the Silver Staining Kit (Nacalai, product #06865−81), and images were captured using the Fusion SL system (Vilber).

### Statistical analysis

All analyses were conducted in R (version 4.4.1, https://cran.r-project.org/). For the estimation, we fitted four-parameter logistic models using the “glm” function, adding a small constant (e.g., 1 × 10^−9^) to zero-dose values to enable logistic regression. Difference in LD_50_ among groups was tested using a likelihood ratio test comparing a null model, which assumes a common LD_50_ across all groups, with an alternative model that allows group-specific LD_50_. This approach enabled us to assess whether the observed variations in LD_50_ were statistically significant, thereby indicating distinct susceptibilities across the tested conditions. Survival analysis was performed using the “survival” package, with a log-rank test for each comparison. Statistical and graphical methods were used to evaluate survival rates and dose–response relationships across various experimental conditions. Kaplan–Meier survival curves were generated using the “survfit” function in the “survival” package, and group differences were assessed using log-rank tests. Dose–response relationships were analyzed using the “drc” package, fitting four-parameter logistic (4PL) regression models via the “drm” function. To ensure model stability, zero-dose values were replaced with a small positive constant (e.g., 1 × 10^−9^). LD_50_ values were estimated using the “ED” function, and confidence intervals were calculated to evaluate their precision. Dose–response curves among groups were compared using likelihood ratio tests between shared-parameter and group-specific models. In cases where model convergence was challenging, modified model specifications were employed with adjusted appropriate parameters to ensure stable fitting. Multiple testing correction was applied using the false discovery rate method via the “p.adjust” function. These analyses were implemented in R (4.4.1), ensuring reproducibility and consistency across all datasets.

## Results

### Pathogenicity of Mucorales can be assessed using the silkworm *B. mori*

**Fig 1:**
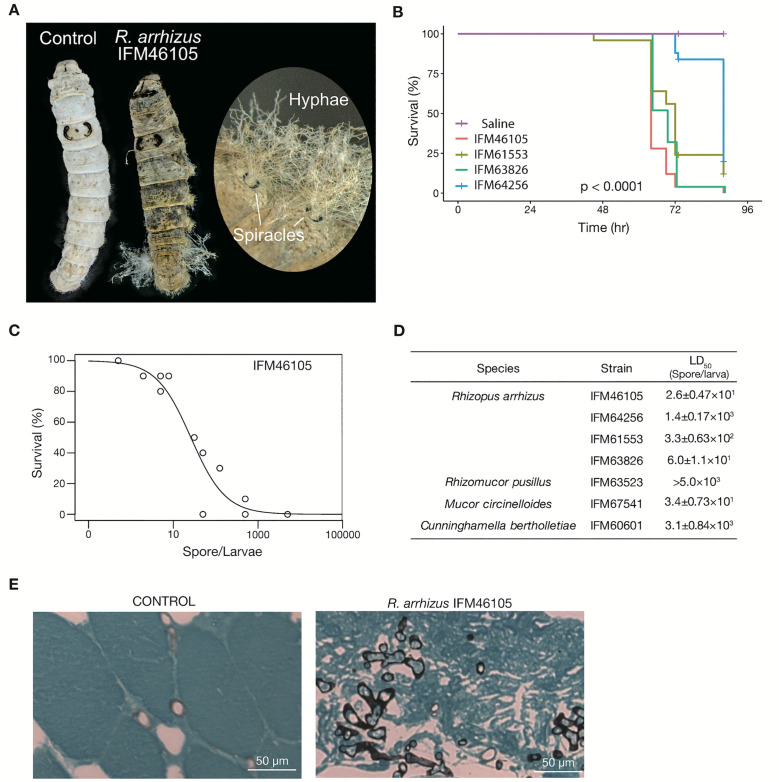
Experimental infection of silkworms with Mucorales. (A) Morphological comparison of control and infected silkworm larvae. The control larva (left) demonstrates typical healthy physiological features, whereas the larva infected with the *R. arrhizus* strain IFM46105 (right) exhibits extensive hyphal growth from the spiracles, indicating severe mycosis. (B) Kaplan–Meier survival curves showing the relationship between time post infection (x-axis) and silkworm survival rate (y-axis) at 27°C. Silkworm larvae (n = 25 per strain) were injected into the hemolymph, with 500 spores/larva of each *R. arrhizus* strain (IFM46105, IFM61553, IFM63826, and IFM64256); the saline-injected control group (n = 15) showed 100% survival. Survival differences among groups were significant (p < 0.0001, log-rank test). For clarity, the IFM63826 survival curve was shifted by +0.5 h horizontally for display purposes only. All statistical analyses were performed on the original, unshifted survival data. (C) Dose–response curve showing the relationship between *R. arrhizus* IFM46105 spore inoculum size (x-axis) and silkworm survival rate (y-axis) at 27°C. The curve fitting suggests a dose-dependent decline in survival, with an estimated LD_50_ (median lethal dose) shown in Fig 1D. (D) LD_50_ of Mucorales toward silkworms varies among strains and species. Pathogenicity differences among *R. arrhizus* strains under infection conditions at 27°C were evaluated by LD_50_. *R. arrhizus* IFM46105 and IFM63826 exhibited a particularly low LD_50_, indicating high virulence relative to *R. arrhizus* IFM64256 and IFM61553. **(E)** Histopathological examination of silkworm tissues. Control larval tissue (left panel) displaying normal tissue architecture, whereas larval tissue infected with *R. arrhizus* IFM46105 (right panel) showed extensive fungal hyphal invasion and associated tissue disruption. The tissue sections were stained using the Grocott’s staining method. Scale bars = 50 µm.

To determine whether the pathogenicity can be assessed using a silkworm-based assay, spores of the *R. arrhizus* strain IFM46105 were inoculated into silkworm larvae and incubated. The infected larvae died, with fungal hyphae protruding from silkworm spiracles—the openings of the tracheal system (Fig 1A). Silkworms infected with different strains of *R. arrhizus* (IFM61553, IFM63826, IFM46105, and IFM64256) showed varying survival rates over time (Fig 1B). The saline control group maintained 100% survival throughout the observation period, whereas all fungal strains caused mortality in silkworms, with significant differences in survival curves (p < 0.0001). Among the tested strains, IFM46105 caused the most rapid decline in survival, followed by IFM63826 and IFM61553, whereas IFM64256 exhibited the least virulence (Fig 1B). The survival rate of the silkworms decreased with increasing dose of Mucorales spores (Fig 1C; Fig S1). The LD_50_ ranged from 26 (strain IFM46105) to 1,400 spores (strain IFM64256) per silkworm, showing up to a 54-fold difference between strains (Fig 1D; Fig S1). Observation of tissues from silkworm larvae infected with IFM46105 (the most virulent strain) revealed hollow hyphae penetrating host tissues, similar to those seen in mucormycosis (Fig 1E, right panel).

Furthermore, we evaluated the infectivity of other Mucorales species using the silkworm-based method. Dose-dependent silkworm lethality was observed in all tested Mucorales fungi, except *R. pusillus* (Fig 1D). When LD₅₀ values were statistically analyzed using generalized linear model and Tukey’s multiple comparison tests, *R. arrhizus* IFM61553 (p = 0.00811) and *R. arrhizus* IFM64256 (p = 0.00247) showed significantly higher LD₅₀ values (i.e., indicating lower pathogenicity) than *R. arrhizus* IFM46105. The LD₅₀ of *R. arrhizus* IFM63826 was slightly higher than that of *R. arrhizus* IFM46105 (p = 0.02421). Collectively, these results suggest that the pathogenicity of Mucorales can be quantitatively evaluated using silkworms.

### The silkworm-based mucormycosis model reflects human clinical risk factors

To examine the human relevance of the silkworm-based infection model, we implemented the risk factors known for mucormycosis infection in human clinical settings in the model. Silkworms were treated with a steroid drug, iron supplement, or iron chelators to simulate immunosuppression, hyperferritinemia, and iron chelator administration, respectively, which increase mucormycosis risk in humans, and their survival following *R. arrhizus* infection was compared to that in untreated silkworms.

**Fig 2:**
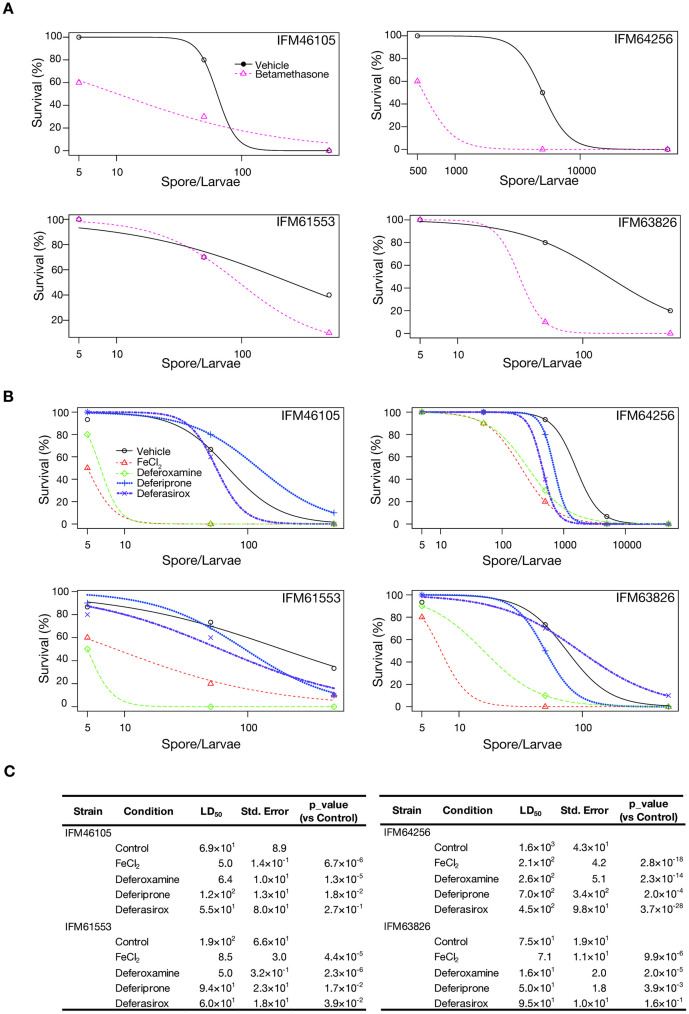
Analysis of risk factors for mucormycosis in the silkworm infection model. (A) Effect of betamethasone on the survival of silkworms infected with *Rhizopus* strains: this series of graphs illustrates the survival percentages of silkworm larvae in response to varying concentrations of spores from different *Rhizopus* strains (IFM46105, IFM64256, IFM61553, and IFM63826). Each plot compares the survival outcomes between larvae treated with 10% DMSO (black circles, solid line) and those treated with 10 mg/mL betamethasone (pink triangles, dashed line). Betamethasone treatment reduced survival relative to 10% DMSO controls. (B) Effect of iron chelators and iron supplementation on the survival of silkworms infected with *Rhizopus* strains: this set of survival curves assesses the effects of vehicle (black), iron (1 mM FeCl_2_, red), and different 1 mg/mL iron chelators (deferoxamine, green; deferiprone, blue; and deferasirox, purple) on the survival of silkworm larvae challenged with spores of *Rhizopus* strains (IFM46105, IFM64256, IFM61553, and IFM63826). The plots reveal the differential impact of these treatments on *Rhizopus* pathogenicity, where iron (FeCl_2_) and an iron chelator deferoxamine significantly enhanced pathogenicity, as evidenced by the left shift of survival curves compared with vehicle treatment (likely because of increased iron availability for fungal growth). In contrast, other iron chelators, such as deferasirox and deferiprone, exhibited a subtle or no effect. (C) Statistical analysis of the effects of iron chelators and supplementation on the survival of silkworms infected with *R. arrhizus* strains: this table presents LD_50_ values (in spores per larva) for silkworms exposed to different treatments, along with standard errors and p-values compared with control conditions. The data are categorized by *R. arrhizus* (IFM46105, IFM61553, IFM63826, and IFM64256) and treatment conditions (control, FeCl_2_, deferoxamine, deferiprone, and deferasirox).

Betamethasone administration enhanced the lethality of all four *R. arrhizus* strains (Fig 2A), suggesting that the silkworm-based mucormycosis model represents immunological involvement in the infection process, similar to that observed in human cases. When an aqueous solution of FeCl₂ was administered to silkworms, the lethality of all four tested *R. arrhizus* strains increased (Fig 2B and 2C). Furthermore, the administration of deferoxamine, an iron chelator that can be utilized by Mucorales [35], enhanced lethality in all four strains of *R. arrhizus* (Fig 2B and 2C). In contrast, the effects of other iron chelators, including deferiprone [35] and deferasirox [32], on lethality varied among the *R. arrhizus* strains. Deferasirox increased silkworm lethality for strains IFM61553 and IFM64256 but had no effect on the lethality of IFM46105 and IFM63826 strains (Fig 2C). These results suggest that steroid administration and iron utilization capacity contribute to the pathogenicity of *R. arrhizus* in silkworms.

### Isavuconazonium prolongs the survival of silkworms injected with *R. arrhizus*

**Fig 3:**
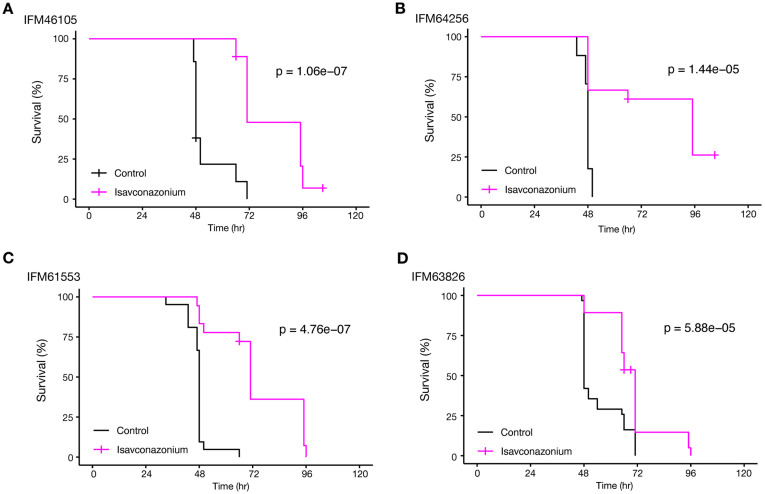
Efficacy of existing antifungal drugs in mucormycosis treatment in silkworms. (A–D) Kaplan–Meier survival curves illustrating the therapeutic effects of isavuconazonium in silkworms infected with various *R. arrhizus* strains. Inoculum sizes were adjusted to 5,000 spores per larva for IFM46105 and IFM63826 and to 50,000 spores per larva for IFM64256 and IFM61553. Survival curves for 5 mM isavuconazonium-treated silkworms (pink lines) showed significant improvements in survival over time compared with the control group (black lines). Panels A–D correspond to infections with IFM46105, IFM64256, IFM61553, and IFM63826 strains, respectively. The respective p-values are indicated in panels.

We investigated whether the first-line mucormycosis drug isavuconazonium, a prodrug metabolized *in vivo* to isavuconazole [36], exhibits therapeutic efficacy in the silkworm-based model. First, the *in vitro* drug susceptibility of each strain to isavuconazole was determined. MIC values of IFM46105, IFM61553, IFM63826, and IFM64256 strains were 22, 2.8, 22, and 2.8 μg/ml (0.050, 0.0063, 0.050, and 0.0063 mM), respectively. *In vivo*, isavuconazonium administration significantly prolonged the survival of all four *R. arrhizus*-infected groups (Fig 3A–3D), confirming its therapeutic efficacy in the silkworm-based model.

### The silkworm-based mucormycosis model enables investigation of the molecular mechanisms underlying Mucorales pathogenicity

**Fig 4:**
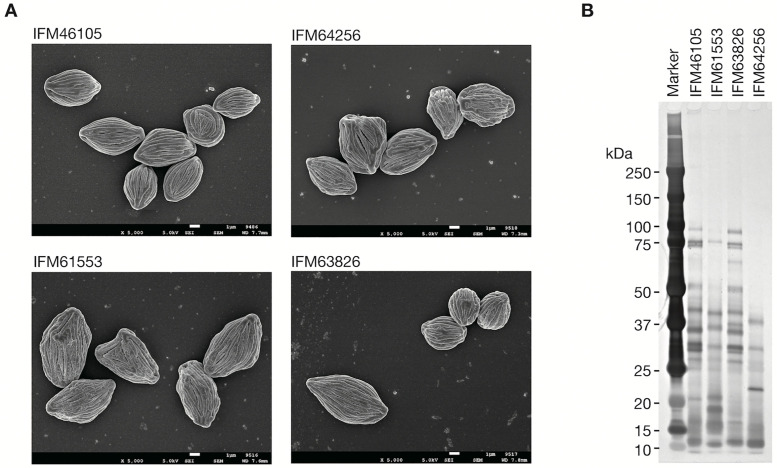
Differences in cell surface proteins among *Rhizopus arrhizus* strains. (A) Scanning electron microscopy (SEM) images of *R. arrhizus* spores: scanning electron micrographs of spore surfaces from four *R. arrhizus* strains (IFM46105, IFM61553, IFM63826, and IFM64256), illustrating uniform overall morphology but distinct microstructural differences. Strains IFM46105 and IFM63826 exhibited smooth linear ridges, whereas IFM61553 and IFM64256 showed irregular surface patterns. These microstructural variations may influence spore adhesion, dispersal, and pathogenic interactions. Each image is displayed with scale bars for reference. (B) Silver-stained SDS-PAGE analysis of cell surface proteins from *Rhizopus arrhizus* strains: this gel electrophoresis analysis highlights notable differences in protein composition among the strains IFM46105, IFM61553, IFM63826, and IFM64256. In particular, high-molecular weight proteins were notably absent in strain IFM64256, which correlates with its observed low pathogenicity toward silkworms. In contrast, highly pathogenic strains IFM46105 and IFM63826 demonstrated a greater abundance of high-molecular weight proteins, suggesting their potential role in enhanced virulence. The molecular weight marker (left lane) facilitates band size comparisons, demonstrating the diversity in protein profiles across strains.

To elucidate the difference in pathogenicity among the four *R. arrhizus* strains, we further investigated the differences in cell surface structures in direct contact with the host. SEM revealed characteristic deep wrinkled, irregular rugby ball-like spores across all *R. arrhizus* strains, consistent with typical *Rhizopus* morphology (Fig 4A). Given that surface adhesion proteins contribute to pathogenicity in other fungi, such as *Candida* and *Aspergillus* [37,38], we analyzed cell surface protein patterns among the four strains with varying pathogenicity toward silkworms. Consequently, high-molecular weight proteins with low electrophoretic mobility were less abundant in the low-pathogenicity strain IFM 64256, whereas they were intermediately abundant in the moderately pathogenic strain IFM61553 and most abundant in the highly pathogenic strains IFM46105 and IFM63826 (Fig 4B). Notably, the two highly pathogenic strains exhibited similar surface protein patterns, suggesting their roles in improved pathogenicity.

## Discussion

In this study, we established the silkworm *B. mori* as a quantitative model for the assessment of Mucorales pathogenicity using LD₅₀ indices. The model was validated using three Mucorales species spanning three genera, including the primary causative agent of mucormycosis *R. arrhizus* and *M. circinelloides* and *C. bertholletiae*. Moreover, we demonstrated its human relevance, showing that the pathogenicity of *R. arrhizus* is enhanced in silkworms upon administration of iron supplements, the iron chelator deferoxamine, and steroid, which simulate the human mucormycosis risk factors of hyperferremia, deferoxamine therapy, and immunocompromised states, respectively. The silkworm model could successfully replicate human mucormycosis pathogenesis and risk factors, enabling quantitative pathogenicity assessment across multiple Mucorales species.

Previous studies have reported that Mucorales species belonging to the genera *Rhizopus* and *Mucor*, which are pathogenic to mammals, exhibit larvicidal activity against silkworms [39,40]. In the present study, we further demonstrated that *Cunninghamella* species possess larvicidal capability against silkworms, expanding our understanding of Mucorales pathogenicity in this invertebrate model. We also demonstrated that infection with *R. arrhizus* can be mitigated by treatment with 5 mM isavuconazonium (203 μg/larva), significantly prolonging larval survival in silkworms. These findings are consistent with those of previous reports by Kurakado *et al*. and Tominaga *et al*., who found that 250 μg/larva posaconazole or 2.5 to 5 μg/larva amphotericin B exerts life-prolonging effects against *R. arrhizus* (formerly known as *R. oryzae*) infection in silkworms [40,41]. Together, these results indicate that silkworms can be used for the evaluation of clinically effective antifungal drugs and for antifungal screening against Mucorales species.

Both iron ions and the iron chelator deferoxamine enhanced the larvicidal activity of all tested strains against silkworms. Given that deferoxamine can be used as a siderophore by Mucorales [35], the utilization of iron ions by Mucorales is essential for the manifestation of pathogenicity in silkworms, as in mammalian hosts. In contrast, while the effects of other chelating agents on Mucorales pathogenicity varied among strains, none demonstrated a pronounced reduction in survival time, as observed with deferoxamine. Notably, deferiprone treatment of silkworms infected with strain IFM46105 prolonged larval survival; this finding was consistent with that of a previous study reporting that deferiprone does not promote iron uptake by Mucorales *in vitro* and prolongs survival in guinea pig mucormycosis models [35]. These results highlight the utility of the silkworm mucormycosis infection model as a valuable platform for screening compounds with therapeutic potential that disrupt Mucorales iron utilization *in vivo*.

In the present study, the silkworm-based model revealed a 54-fold difference in LD₅₀ among *R. arrhizus* strains. In contrast, murine intravenous mucormycosis infection models have reported over 100-fold differences in LD₅₀ values among Mucorales fungal species, ranging from 0.4 × 10³ spores/mouse for *R. cohnii* to >126.1 × 10³ spores/mouse for *M. alternans* [42]. However, no intraspecies difference has been reported. Differences in Mucorales pathogenicity at both the species and strain levels can be attributed to factors such as spore germination capacity, resistance to host immune responses, and variations in CotH gene copy numbers [43]. In particular, comparative genomic and functional analyses highlighted that the number of CotH genes differs among species and contributes to variations in virulence observed in animal infection models [44]. Comparison of the patterns of spore surface proteins among these strains revealed that the highly pathogenic group (i.e., IFM46105 and IFM63826, sharing similar genomic structures) exhibited a distinct surface protein pattern compared with other strains (Fig 4B). Notably, surface protein analysis revealed that the highly pathogenic strains (IFM46105 and IFM63826) expressed higher quantities of high-molecular weight surface proteins in the 50–100 kDa range, whereas the low pathogenic strains (IFM64256 and IFM61553) showed less quantities of these proteins. Regarding the involvement of cell surface proteins in pathogenicity, a closely related species *Rhizopus delemar* expresses a 64-kDa (molecular weight excluding the signal peptide sequence) cell surface spore coat protein homolog 3 (CotH3), which is a virulence factor essential for adhesion to host cells through the mammalian GRP78 protein [7]. Antibodies against CotH3 protein prolong survival in murine mucormycosis models [45]. The silkworm genome encodes a GRP78 homolog with 81% sequence identity to its mammalian counterpart, suggesting that this invertebrate model has the potential to replicate the molecular process of host–pathogen interactions that occur in mammalian mucormycosis. In addition to CotH3, the CotH7 protein (ca. 65 kDa) of *R. delemar* interacts with host integrin β1 (also conserved in silkworms [46]) and contributes to cell invasion [47,48]. These findings suggest that the virulence factors CotH3 and CotH7 (fungal proteins) play a critical role in Mucorales pathogenicity in silkworms possibly through interactions with conserved host receptors such as GRP78 and integrin β1, similar to their roles in mammalian hosts.

In conclusion, our study highlights the value of the silkworm infection model for quantitative pathogenicity assessment and antifungal efficacy testing against Mucorales. By expanding its application to high-throughput screening, we open new avenues for the rapid identification of effective antifungal agents. This approach holds great promise for overcoming current challenges in mucormycosis treatment, especially in the face of rising antifungal resistance and limited therapeutic options. Future research leveraging this model may yield important insights in mucormycosis pathogenesis and accelerate the development of effective therapies to combat this life-threatening infection.

## Supporting information

Fig S1 Dose–response curve showing the relationship between *Rhizopus arrhizus* IFM64256, IFM61553, and IFM63826; *Mucor circinelloides* IFM67541; and *Cunninghamella bertholletiae* IFM60601 spore inoculum size (x-axis) and silkworm survival rate (y-axis) at 27°C. (PDF)

Table S1 Comparison of silkworm survival between the two dilution methods. (PDF)
