# Supplementary material for: A human-relevant alternative infection model for mucormycosis using the silkworm Bombyx mori
Source: PLoS One. 2025 Sep 25;20(9):e0333476. doi: 10.1371/journal.pone.0333476 (PMC12463241; doi:10.1371/journal.pone.0333476)
Supplement: Fig S1 — (PDF) [file pone.0333476.s001.pdf]

Rhizupus arrhizus

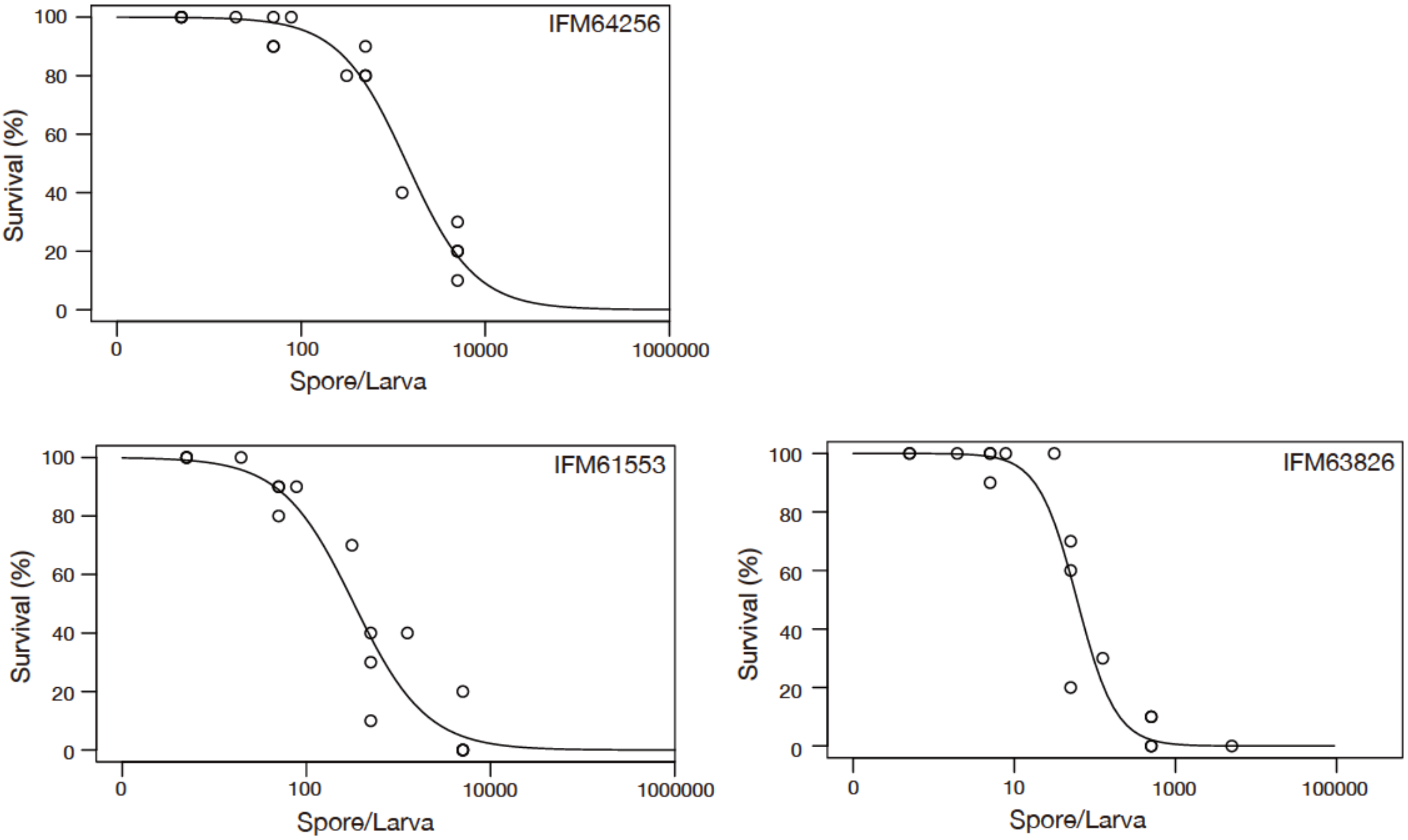

Mucor circinelloides

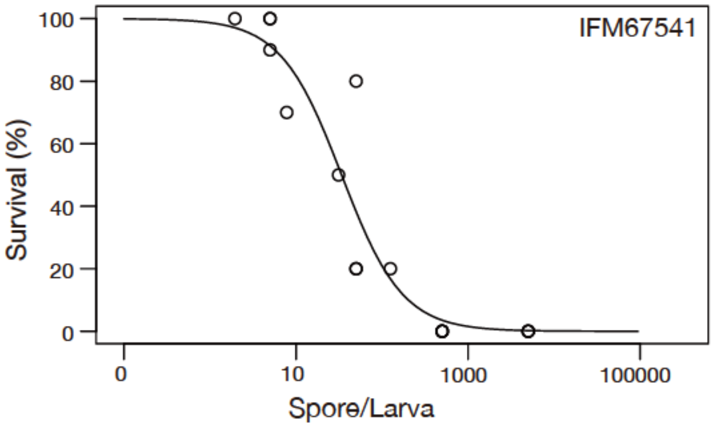

Cunninghamella bertholletiae

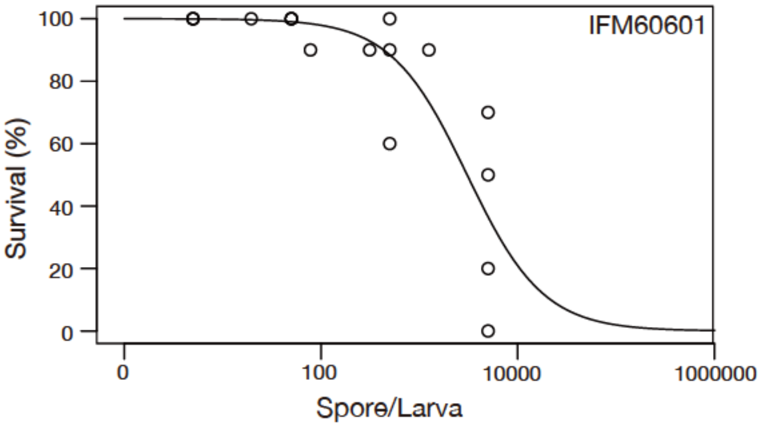

Figure S1

Figure legend S1

The dose-response curve shows the relationship between *R. arrhizus* IFM64256, IFM61553, and IFM63826, *Mucor circinelloides* IFM67541, and *Cunninghamella bertholletiae* IFM60601 spores inoculum size (x-axis) and silkworm survival rate (y-axis) incubated at 27°C.
