# Supplementary material for: A human-relevant alternative infection model for mucormycosis using the silkworm Bombyx mori
Source: PLoS One. 2025 Sep 25;20(9):e0333476. doi: 10.1371/journal.pone.0333476 (PMC12463241; doi:10.1371/journal.pone.0333476)
Supplement: Table S1 — (PDF) [file pone.0333476.s002.pdf]

Injectioon dose spore/larvae		Day 1		Day 2		Day 3				Day 4	
		21 (hr)	33.5	46	53.5	69	72	75	78	94	101
0 (Control)	plastic	5	5	5	5	5	5	5	5	5	5
	glass	5	5	5	5	5	5	5	5	5	5
780	plastic	5	5	5	4	1	1	1	0	–	–
	glass	5	5	5	4	1	1	1	0	–	–
195	plastic	5	5	5	5	4	3	3	3	1	0
	glass	5	5	5	5	3	3	2	2	0	0

Table S1

Table S1 Comparison of silkworm survival between two dilution methods

Survival of infected silkworms injected using plastic and glass dilution vessels and syringes. The data demonstrates no substantial difference between the two methods.

Conidia suspensions of *Rhizopus arrhizus* IFM46105 (2×10^8 conidia/mL) were diluted in either plastic tubes (FALCON 50 mL Polypropylene Conical Tube) or glass vials (Mighty Vial No.5 (19 mL)). Fifth-instar day 2 silkworm larvae were injected with 50 µL of the conidia suspensions into their hemolymph using either plastic syringes (TERUMO 1 mL syringe with 27-gauge needle) for plastic tube-prepared suspensions or glass syringes (TOP Glass Syringe 0.5 mL Interchangeable) for glass vial-prepared suspensions. The infected larvae were maintained in an incubator at 27°C. The number of surviving silkworms was counted and survival times were measured.
